# Beta-Adrenergic Agonists, Dietary Protein, and Rumen Bacterial Community Interactions in Beef Cattle: A Review

**DOI:** 10.3390/vetsci10090579

**Published:** 2023-09-18

**Authors:** Alison P. Pfau, Elizabeth A. Shepherd, M. Gabbi Martin, Sophia Ascolese, Katie M. Mason, Amanda M. Egert-McLean, Brynn H. Voy, Phillip R. Myer

**Affiliations:** Department of Animal Science, University of Tennessee, Knoxville, TN 37996, USA

**Keywords:** beta-adrenergic agonists, microbiome, protein, ractopamine hydrochloride, rumen

## Abstract

**Simple Summary:**

The beef industry faces the challenge of increasing the supply of high-quality protein to an ever-growing global population. Improving nutrient use efficiency is important to enhancing the sustainability of the beef industry and its environmental footprint. Over the past few decades, advances in dietary innovations and feed supplements have helped to improve feed efficiency in cattle. However, focusing on the rumen microorganisms stands to rapidly advance ruminant nutritional physiology, as the microbes enable ruminant animals to utilize plant-derived feed sources by processing them through microbial-driven fermentation. To address common feed and management practices and their impact on the rumen microbial community, this review addresses the interactions among beta-adrenergic agonists, protein level and source, and the ruminal microbiome. Advancing our understanding of feed and management practices and their association with the rumen microbiome will help to sustainably improve beef cattle performance.

**Abstract:**

Improving beef production efficiency, sustainability, and food security is crucial for meeting the growing global demand for beef while minimizing environmental impact, conserving resources, ensuring economic viability, and promoting animal welfare. Beta-adrenergic agonists and dietary protein have been critical factors in beef cattle production. Beta-agonists enhance growth, improve feed efficiency, and influence carcass composition, while dietary protein provides the necessary nutrients for muscle development and overall health. A balanced approach to their use and incorporation into cattle diets can lead to more efficient and sustainable beef production. However, microbiome technologies play an increasingly important role in beef cattle production, particularly by optimizing rumen fermentation, enhancing nutrient utilization, supporting gut health, and enhancing feed efficiency. Therefore, optimizing rumen fermentation, diet, and growth-promoting technologies has the potential to increase energy capture and improve performance. This review addresses the interactions among beta-adrenergic agonists, protein level and source, and the ruminal microbiome. By adopting innovative technologies, sustainable practices, and responsible management strategies, the beef industry can contribute to a more secure and sustainable food future. Continued research and development in this field can lead to innovative solutions that benefit both producers and the environment.

## 1. Introduction

Ruminants have the capability to utilize different resources or foodstuffs and transform them by different processes into high-quality protein for human consumption. To meet the growing protein demand of humans, beef producers must implement technologies to grow beef cattle efficiently while considering and managing animal welfare [1]. An objective of beef cattle production is to increase muscle mass, which can be accomplished through improvements in feed efficiency. For this reason, the components and nutrient composition of finishing cattle diets have historically been altered by increasing the use of byproducts from the corn milling industry, as corn grain has noted advantages in improving the efficiency of growth [2]. These byproducts result in diets with an excess supply of dietary protein [3]. Research has shown limited enhancements in animal performance when the crude protein (CP) concentration in finishing diets was greater than 13% [4,5] and if the CP was greater than 15.5% (by the combination of protein degradability), results demonstrated a possible metabolic cost related with ammonia detoxification from the liver. These results indicate protein degradability likely performs a critical role in animal performance [6].

With continuing interests regarding the improvement of lean mass yield, producers and researchers continue to develop new or improved technologies to enhance the performance of finishing cattle. In the US, technologies include growth-promoting steroidal implants and beta-adrenergic agonists, which are generally fed to beef cattle between the last 70 and 120 days before harvest to increase muscle growth [7]. Utilization of implants in the beef industry has been common practice in the US since 1956, with the approval of diethylstilbestrol (DES). Since then, many varieties of implants have been developed with the purpose of maximizing productivity and minimizing negative costs to meat quality. Beta-adrenergic agonists and their respective host receptors were introduced in the 1970s as a repartitioning agent, redirecting energy to protein accretion and reducing lipid deposition [8]. The economic benefits of the beta-adrenergic agonists made them an excellent tool for feedlot consulting nutritionists, who reported that 85% of their customers used beta-adrenergic agonists during the finishing period [3]

In 2003, ractopamine hydrochloride (RAC) [9], a beta-adrenergic agonist (synthetic catecholamine) marketed under the commercial trade name Optaflexx (Elanco Animal Health. Greenfield IN), was approved for use in cattle feed in the United States at a rate of 70–430 mg/animal/day during the last 28 to 42 days prior to harvest [10,11]. Years later, zilpaterol hydrochloride (Merck Animal Health, Summit, NJ) was accepted for use in cattle feed in the United States at a proportion of 8.3 mg/kg on a dry matter basis in a complete feed for the last 20 to 40 days prior to harvest. However, in contrast with ractopamine hydrochloride, which has a zero-day withdrawal, zilpaterol hydrochloride requires a withdrawal period of three days to ensure animals are without drug residues. Ractopamine hydrochloride has been used in cattle to increase muscle mass, showing greater results when it is included in the diet in parameters such as final body weight (BW), carcass weight, and animal performance. In finishing steers, the dietary supplementation of RAC resulted in 7.3 to 10.1 kg greater final BW [12,13], whereas in finishing heifers, animals were 8.3 to 10.6 kg greater in final BW. RAC also improved the average daily gain (ADG), resulting in 16 to 32% greater ADG compared to animals without the supplementation of RAC [10,14,15]. It should be noted, however, that the use of beta-adrenergic agonists in livestock is not approved worldwide, such as in the European Union.

Studies investigating the interaction between RAC and dietary protein have demonstrated animals fed diets high in ruminally degradable protein in combination with RAC improved the response of RAC [16,17]. The interaction between RAC and dietary protein has led researchers to examine rumen microbiota. Naturally occurring catecholamines have been shown to affect certain types of microorganisms, including bacteria. Supplementation with RAC altered proteolysis processes in the rumen, affecting the availability of the rumen microbiota to utilize ruminally degradable protein [16]. The ratio of protein-degradable fractions (RDP and RUP) could influence the response of RAC [16]. Therefore, this review aims to explore the interaction among different protein concentrations, beta-adrenergic agonists, and ruminal communities of beef cattle to evaluate the impact of beta-adrenergic agonists, dietary protein, and microbiome interactions to improve animal performance and efficiency.

## 2. Beta-Adrenergic Agonists

To accelerate animal growth by improving lean tissue accretion and address the global demand for additional beef, livestock production has commonly used beta-adrenergic agonists. Beta-adrenergic agonists (β-AA) are phenethanolamine compounds with similar physical and pharmacological characteristics to endogenous catecholamines, such as epinephrine and norepinephrine [18]. β-AA have been shown to improve feed efficiency, increase the rate of gain, and decrease the deposition of fat in the carcass through stimulation of adrenoreceptors situated on the membrane into muscle and adipose tissue [19,20,21]. The structure of all *β*-AA conforms to a six-membered aromatic ring, a hydroxy group bound with a β carbon, a charged N in the ethylamine side chain, and an adjacent R group to the aliphatic N, which is required for biological activity [22]. However, some differences in substitution of the aromatic and the R group, which are essential for subsequent activity, can contribute to the affectation of tissue longevity, metabolism, and affinity of beta-adrenergic receptors [22] while also preventing rapid deactivation of the *β*-AA [22]. Moreover, proper functioning of β-AA requires the presence of the aliphatic amino group. Alkaline pKa of these amino groups permits them to exist in a protonated state in different tissues at physiological pH and ionized at the beta-adrenergic receptors [22].

**Figure 1:**
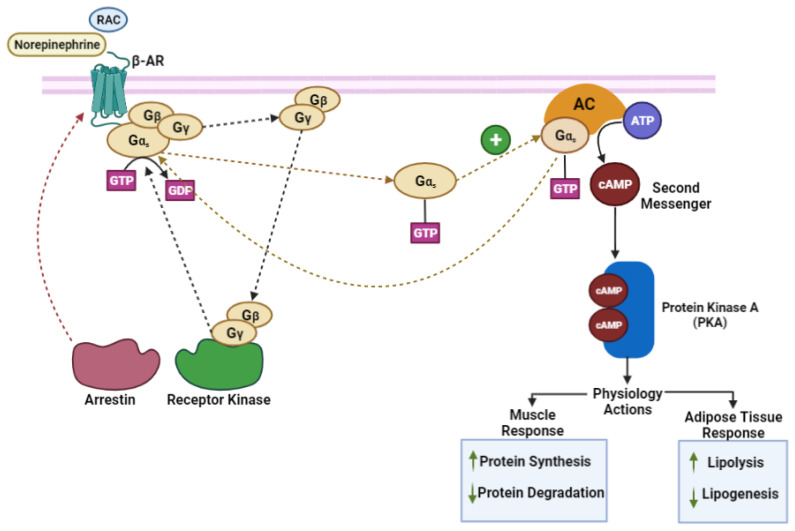
Mechanism of action of beta-adrenoreceptors. (RAC) ractopamine hydrochloride, (*β*-AR) beta-adrenergic receptor, (G_α_, G_β_, G_γ_) Gs protein, (AC) Adenylyl Cyclase enzyme, (ATP) Adenosine Triphosphate (cAMP) Cyclic Adenosine 3′,5′- Monophosphate. (Adapted from [23,26]).

Adrenergic receptors (*α* and *β*-AR) are members of a complex family of G-protein-coupled receptors (GPCR). Adrenergic receptors are positioned in the plasma membrane in mammalian cells, which contain seven hydrophobic membrane-spanning regions with three internal and external segments associated with the N-terminus and C-terminus [23,24]. The C-terminus functions to regulate phosphorylation but is inactivated by phosphorylation at ring 3 located in the G proteins [23]. β-AA bind to *β*-AR, thereby activating G proteins, which stimulates the α subunit of the G protein to dissociate from the γ and *β* subunits and activates the Adenylyl Cyclase enzyme by the binding of GTP. The reaction produces cyclic Adenosine Monophosphate (cAMP), one of the main intracellular signaling molecules regulating gene transcription and protein expression. The action of cAMP requires binding with protein kinase A (PKA) phosphorylating intracellular proteins, such as, hormone-sensitive lipase, an enzyme for adipocyte triacylglycerol degradation [25] (Figure 1).

**Figure 2:**
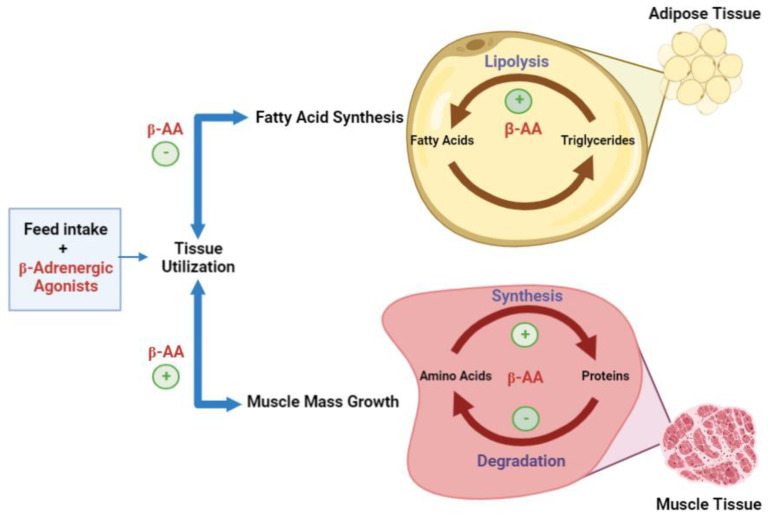
Proposed mode of action of beta-adrenergic agonists in the accretion of muscle growth and adiposity reduction (adapted from [23]).

The phosphorylation helps to increase the transcriptional action of the cAMP response element-binding protein (CREB), which is phosphorylated prior to the action of PKA. The transcriptional activity of CREB provides the tools for β-AR agonists to facilitate the transcription of a number of genes in mammalian cells. However, enzymes such as acetyl-CoA carboxylase and long-chain fatty acid biosynthesis enzymes can be inactivated by phosphorylation [25]. Through stimulation of hydrolysis or lipolysis and the inhibition of de novo fatty acid biosynthesis, *β*-AA can decrease the adipose tissue accretion and contrarily produce the increase of muscle mass by the inhibition of protein turnover, encouraging myofibrillar protein synthesis (Figure 2).

Historically, *β*-Adrenergic receptors have been classified into different subtypes, represented as *β*1, *β*2, and *β*3 [27]. However, classifying *β*-Adrenergic receptors has been difficult due to the selectivity, mechanisms for signal transduction, differences in ligand binding affinity, physiological effects, and differences in distribution across species and tissues [25,27]. For example, human tissues have a ratio of 80:20 *β*1:*β*2-AR versus rat tissues with a 15:85 *β*1:*β*2-AR ratio [28,29]. Also, abundances of *β*-AR subtype mRNA transcripts in different tissues of porcine existed, indicating greater ratios of *β*1 over *β*2 in subcutaneous adipose tissue (81:19), skeletal muscle (59:41), heart tissue (72:28), and lung tissue (58:42) [29]. Despite the study of the characterization of β-AR subtypes in different species, such as humans [30] and porcine [25,31], information regarding *β*-AR in bovine tissues is still relatively limited. Some reports of proportions of the *β*-AR subtypes in bovines have shown that more than 99% of *β*2-AR are located in the skeletal muscle and around 90% in the adipose tissue [32].

One of the important characteristics of *β*-AA is the rapid absorption after an oral administration [8]. When experimentally dosing dogs, rats, and swine with *β*-AA, concentrations peaked in the plasma after 0.5–2 h, and total elimination was observed 6 to 7 h after the initial administration [33]. Additionally, evidence suggests *β*-AA are absorbed rapidly in the gastrointestinal tract through passive diffusion due to the neutrality of the pH, which prevents the formation of cations at the phenethanolamine nitrogen and helps the absorption through the intestinal mucosa [22,34]. However, the information about the site of absorption in ruminant species is still limited.

## 3. Mechanism of Action

### 3.1. Effects on Skeletal Muscle Deposition

Supplementation with *β*-AA impacts growth by increasing the accretion of skeletal muscle via muscle hypertrophy and directly reducing lipolysis [35]. Accretion of skeletal muscle results in increased synthesis of protein and/or lower degradation of protein, improving animal muscularity [25,33]. *β*-AA benefits can be short-term. Desensitization of *β*-AR in experiments with rats demonstrated weight gain over 7 days but decreased to zero by day 14 [36]. Regarding the desensitization of *β*-AR in ruminants [37], a similar pattern was shown with pigs, where the response of *β*-AA was positive during the first week, but the response declined to zero by week 7 [38]. These data suggested that the down-regulation of *β*-AR in adipose tissue could prevent the complete expression of receptors without changing the rate of adipose tissue accretion [39]. Thus, the increase in skeletal muscle, without increasing adipose, has attracted producers to the use of *β*-AA in the final finishing phase of feeding in animal production [39].

Animals supplemented with *β*-AA result in greater blood flow into the muscle, allowing a greater flow of nutrients and improving the efficiency of the muscle cell growth [25,40]. In addition to muscle hypertrophy, studies have demonstrated *β*-AA administration can also increase muscle fiber diameter, as well as affect the different muscle fiber types, such as myosin heavy chain (MHC) type I and II. According to, NRC [35], *β*-AA action increases the growth of type II fibers compared to type I fibers. However, during supplementation with ractopamine hydrochloride (RAC) in cull beef cows, type I fibers increased in diameter, but no response occurred in type II MHC [41].

Increased muscle growth, however, may cause negative effects, as shown in reduced marbling scores and elevated beef toughness, producing an increase in glycolytic fiber types [40,42]. It has been proposed that insulin growth factor-I (IGF-I) could be implicated in skeletal muscle hypertrophy with the presence of *β*-AA due to decreased degradation of protein and increased protein synthesis [23]. An experiment with lambs fed 10 ppm of cimaterol (β-AA) resulted in a reduction of the IGF-I levels by 46.5% at day 42 and 21.5% at day 84 in comparison with control animals [43]. In Holstein animals supplemented with RAC, longissimus muscle (LM) IGF-I mRNA concentrations increased compared to concentrations observed with animals in the control [16]. Additionally, IGF-I is known to stimulate the division of muscle satellite cells by mitosis, which assists postnatal muscle hypertrophy. Therefore, understanding the activity of IGF-I in satellite cell proliferation during the stimulation of *β*-AR by the *β*-AA in skeletal muscle hypertrophy is necessary [23].

### 3.2. Effects on Protein Accretion

Protein synthesis increases in porcine skeletal muscle when supplemented with RAC [44]. RAC can induce muscle protein accretion, enhance protein synthesis, and inhibit protein degradation [23]. Although essential, protein accretion is recognized as an unproductive process that is responsible for approximately 20% of total outflow energy in livestock species during growth [45]. The accretion of protein is divided into two catabolic and anabolic processes, where *β*-AA are involved via different pathways [8]. Due to the presence of *β*-AA in both catabolic and anabolic protein processes, the activity of the proteins involved in the degradation by the calpain system increases [8]. The activity of CREB activation also assists in the reduction of protein degradation by the increase of calpastatin production, directly inhibiting calpain proteases [46].

**Figure 3:**
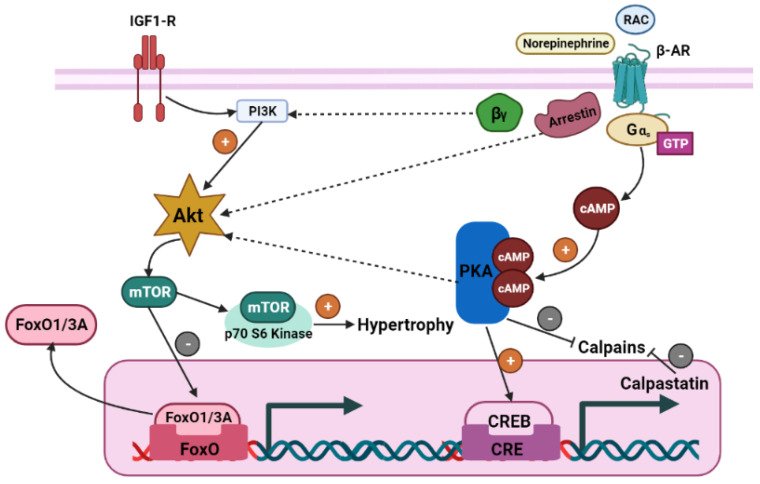
Possible mechanisms of Akt activation by beta-adrenoreceptors signaling that causes myofiber hypertrophy. (RAC) ractopamine hydrochloride, (*β*-AR) beta-adrenergic receptor, (Gα, Gβ, Gγ) Gs protein, (ATP) Adenosine Triphosphate, (cAMP) cyclic Adenosine Monophosphate, (PKA) protein kinase A, (Akt) protein kinase B, (CREB) cAMP response element-binding protein, (FoxO) Forkhead box transcription factor, class O, (IGF-1) insulin-like growth factor-1, (PI3K) phosphatidylinositol 3-kinase, (mTOR) mammalian target of rapamycin. (Adapted from [26]).

Activation of protein kinase B (Akt) signaling targets affects protein synthesis. One such target is mammalian rapamycin (mTOR), which increases protein synthesis by the activation of the ribosomal protein s6 kinase (p70^s6k^) [8]. Ribosomal protein s6 kinase is correlated with elongation and translation and indirectly implicated in the activation of an eukaryotic translation initiation factor (eIF4E), which has an important role in the initiation of protein translation [47]. Another important function of Akt is the inhibition of the protein breakdown process by phosphorylation and inactivation of forkhead box O, which is a transcription factor necessary for E3 ubiquitin ligases [48,49]. Other mechanisms involving the activation of the Akt include the *β*-arrestin, phosphatidylinositol 3- kinase (PI3K), and the initiation of cAMP response element-binding protein (CREB) [50] (Figure 3). These mechanisms of Akt activation by beta-adrenoreceptors signaling help to cause myofiber hypertrophy.

In lambs experimentally supplemented with *β*-AA, CREB and Akt increased calpastatin production and reduced the activity of calpain [51]. Similarly, greater calpastatin was reported in steers supplemented with *β*-AA [52,53]. Regardless of the positive effects of β-AA signaling on the target activity, it is important to understand that the principal effect in protein accretion is directed by the increase of muscle hypertrophy without affecting the myonuclei. Likewise, various studies reported cell proliferation increased; however, the fusion of the satellite cells did not show the same response [54].

## 4. Effects of Catecholamines and Beta-Adrenergic Agonists on the Microbiome

### 4.1. Rumen Bacteria

The rumen is a continuous fermentative ecosystem that provides an ideal anaerobic environment to maintain variable populations of microorganisms [55]. Microbial communities in the rumen include bacteria, archaea, as well as eukaryotic protozoa and fungi. Bacteria and protozoa represent 90% of the total microbial biomass [56]. Rumen microbial populations interact through various biotic relationships such as mutualism (benefits for both microorganisms) and commensalism (benefits for one without influencing the other), allowing the ruminant to obtain the essential nutrients for its nutrition through microbial fermentation processes [57]. Because of the reliance on microbial populations within the rumen for animal health, the rumen is often considered the exemplary cooperative symbiotic system of animal–microbial symbioses, where microbes have been represented as endosymbionts in the course of evolution [58]. Microbial communities in the rumen are traditionally characterized by their physiological, morphological, and ecological differences among them. However, the majority have the capacity to break down, ferment, and/or store polysaccharides and proteins derived from plants [59].

Microbiota in the rumen provide stability to the rumen ecosystem through resilience and functional redundancy. Rumen stability increases microbial adaptation and acclimation in terms of dietary changes or management approaches [56,60]. Ruminants depend on microbial degradation of plant substrates, which is due to the metabolic activity of bacteria species, protozoa, and fungi [61]. Bacteria have been divided into five subgroups based on the interaction with food particles: (1) free bacteria carried in the rumen liquid phase, (2) bacteria weakly related to feed particles, (3) bacteria firmly attached to feed particles, (4) bacteria connected with rumen epithelium, and (5) bacteria adhered to the surface of protozoa or fungal sporangia [62,63].

Peptide fermentation relies on bacteria such as *Bacteroides amylophilus*, *Bacteroides ruminicola*, *Butyrivibrio fibrisolvens,* and *Streptococcus bovis* [64]. Up to 38% of total ruminal bacteria are proteolytic, with at least three microbial proteases: cystine-protease, serine-protease, and metallo-proteinase [58,65]. Ammonia-producing bacteria, such as *Prevotella ruminicola*, *Bacteroides ruminicola*, *Megasphaera elsdenii*, *Selenomona ruminantium,* and *Butyrivibrio sp.*, obtain ammonia from urea hydrolysis and protein deamination. This group of bacteria represents 5% of the total population of the rumen. *Prevotella ruminicola* is one of the most important bacteria in this group, as it is the largest ammonium producer in the rumen [65]. Maximum levels of ammonia in the rumen are reached two hours after feed ingestion, coinciding with the maximum growth of proteolytic bacteria [66]. Bacterial protein is synthesized by total or partial degradation of crude proteins, amino acids, or NH_3_ from the diet [67]. Microbial crude protein (MCP), rumen undegradable protein, and endogenous crude protein play a part in the passage of metabolizable protein to the small intestine to be absorbed and utilized by the animal [68].

Proteolysis of protein in the rumen varies due to characteristics of the protein, such as solubility, structure, animal intake, and feedstuff size [69]. The proteolytic activity of microorganisms in the rumen is around 75%, as these enzymes interact in different digesta fractions of the rumen [70].

### 4.2. Protein Degradation in the Rumen

Requirements of CP for finishing cattle are between 12.5% and 13% of dry matter [71]. However, recent studies have suggested greater levels of protein (13.5%) in the diet for finishing cattle, with the use of nitrogen sources such as cottonseed meal, soybean meal, grain coproducts, and urea [72]. In general, all the proteins contained as components of animal feed are recognized to have a certain “pass-through” effect percentage. A greater percentage of these are degradable in the rumen, and therefore, a lower percentage are usable (digested and absorbed) directly in the small intestine. Highly degradable proteins in the rumen can be converted into NH_3_-N, regardless of their quality. Ammonia is an important substrate for ruminal bacterial protein production, which then passes into the intestine as a natural, high-quality pass protein source to be digested and absorbed by the animal [73].

**Figure 4:**
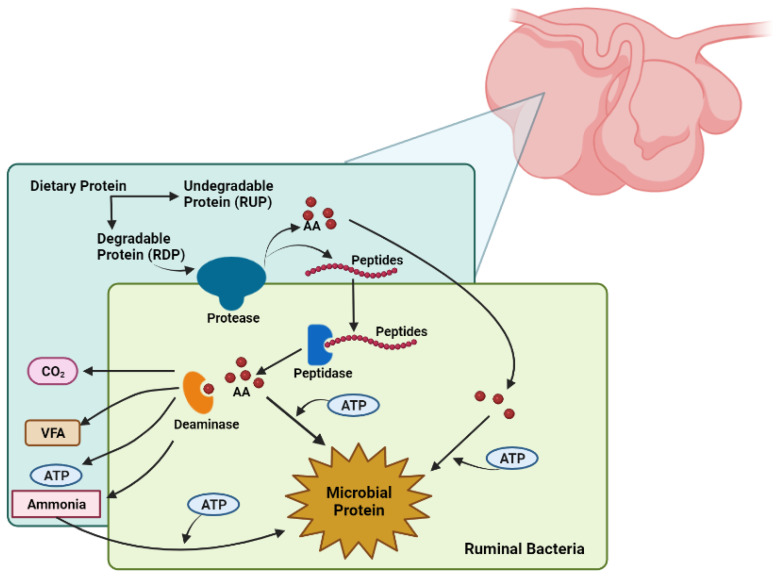
Schematic representation of protein degradation process in the rumen. (Adapted from [26]).

Crude protein in ruminants is divided into two important components of protein: rumen-degradable protein (RDP) and rumen-undegradable protein (RUP). These types of proteins have separate functions. RDP offers a combination of peptides, free amino acids, and ammonia, which is important for microbial growth and the synthesis of microbial crude protein (MCP). This MCP provides the majority of amino acids that pass to the small intestine. RUP is the secondary source of absorbable amino acids in the animal [68]. When the animal consumes RDP, it is degraded by the microbes in the rumen, whereas RUP will escape the reticulorumen, move directly to the abomasum and the small intestine for post-ruminal digestion, and be absorbed by the animal [26]. With regard to RDP, microbial communities can start the absorption of feed particles and begin the breakdown of the peptide bonds of CP by the use of proteases [74]. By degrading CP into peptides or free amino acids, this end product of degradation will be introduced to the microbe. Further, if energy is available, these substrates will be used for the synthesis of MCP [26]. Nevertheless, if the energy required for this process is not available or RDP is provided in greater proportions than the rumen microbial capacity for microbial CP synthesis, the excess protein and amino acids will be deaminated and fermented, producing volatile fatty acids (VFA, such as acetate, propionate, and butyrate) and ammonia (Figure 4). Microbial ammonia is then expelled and released into the rumen for absorption through the rumen wall and further into the bloodstream; in the liver, the overflow of ammonia is converted to urea and excreted in the urine [26,75]. Conversely, when the concentration of ruminal ammonia is low, urea is recycled by two different pathways, via the rumen wall or via the saliva, with the purpose of providing an extra source of N when the dietary protein is limited [75]. The accumulation of amino acid intake may be a limiting factor of protein degradation, suggesting that the manipulation of protein degradation can be completed by the proteolysis modulation and by some modifications in peptidolysis and deamination [26].

Ammonia, as a principal source of nitrogen for ruminal bacteria growth, can affect protein degradation in the rumen and directly negatively affect the synthesis of MCP due to a lower supply of ATP from higher degradable protein in the rumen compared to digestible carbohydrates [76]. However, it is important to prevent free ammonia in the blood because it can potentially result in toxicity and could be dangerous to the animal [3]. In agricultural production, the use of feed-added nitrogen (principally in the form of protein) in animal diets has been inefficient. Addition of feed nitrogen (N) results in increased excretion of ammonia [56]. For optimal productivity, nutritionists have the responsibility of creating alternatives to waste N disposal, improving protein efficiency and use of N, and aiming to reduce feed costs per unit of lean tissue and the implementation of other nutrients in the diet that will enhance production [68].

Byproducts of corn milling are often used in finishing cattle diets and can include corn gluten feeds and distiller grains, which include over 30% crude protein (CP). The proportion of those byproducts in the diets of finishing cattle is greater than 50% to substitute high-energy feedstuffs [72]. Urea is an additional source of non-protein nitrogen (NPN) offered in diets as an alternative source of rumen-degradable protein. Urea can be transformed in the rumen by the ruminal microorganisms into microbial CP [26]. However, in combination with grain byproducts, NPN sources may increase the concentration of dietary CP, depending on the level of inclusion, as grain byproducts contain an excess in CP (over 30%), which exceeds the requirements for CP [77].

### 4.3. Microbial Protein Synthesis

The protein produced by ruminal microbes represents the main source of amino acids and approximately 85% of the total absorbable protein [68] in the ruminant diet. The availability of MCP in the rumen depends on the characteristics of the nutrients in the diet, such as the amount of carbohydrates and proteins and the nutrient-use efficiency of microorganisms. Synthesis of MCP requires ATP for microbial maintenance and growth and requires peptides, amino acids, and ammonia to be used in MCP [68]. Depending on the amount of RUP in the diet of beef cattle, MCP can represent approximately 50% of the metabolizable protein [77].

Feedstuffs play an indispensable role in microbial protein synthesis because synthesis decreases in animals fed high-concentrate diets due to the lower pH in the rumen [77]. When the pH in the rumen drops below 6, the efficiency of microbial enzymes decreases significantly, and bacterial growth experiences a sharp decline [78]. Rumen pH also decreases with low-quality forages due to the slow degradation of carbohydrates [77]. Higher levels of non-structural carbohydrates decrease the concentration of ammonia in the rumen, stimulating microbial protein synthesis [79] and the utilization of nitrogen by the microbes is more efficient. Increasing dietary pectin, cellulose, and hemicellulose increased concentrations of ammonia, and as a result, microbial protein synthesis decreased [80]. Importantly, microbes in the rumen are specialized with regard to their fermentation of structural carbohydrates and non-structural carbohydrates, utilization of ammonia, and fermentation of amino acids and peptides as a primary source of nitrogen [81]. To improve the rumen environment and growth of bacterial species, dietary alternatives can be recommended regarding a mixture of dietary components (e.g., forages and concentrates), which can also increase microbial protein synthesis [82]. Indeed, a proposed strategy for enhancing the utilization of rumen-degradable protein and improving the rate and efficiency of microbial growth involves synchronizing the supply of energy and nitrogen sources to rumen microorganisms [83,84]. The extent to which energy and nitrogen are released can impact microbial protein synthesis, especially when the rumen is supplied with diets high in fermentable carbohydrates. However, limited work has been conducted examining the rumen microbiome response to the synchronism between the availability of N and energy in vivo. In a simulated rumen system, research has demonstrated that the degree of synchronization can influence the bacterial community and enzyme activities of ammonia integration [85]. When using infusions of maltodextrin for 0 to 6 h post-feeding, for 6 to 12 h post-feeding, or continuously through the day (0 to 12 h post-feeding infusion), bacterial genera and metabolites/enzymes were impacted. For example, *Fibrobacter* and *Ruminobacter* relative abundances were decreased in the 6 to 12 h post-feeding infusion compared to the other infusions. Further, microbial protein synthesis showed a positive correlation with the activity of GDH (glutamate dehydrogenase) and the relative abundance of *Fibrobacter* [85]. Beyond the numerous results noted, the researchers demonstrated that the degree of synchronization and bacterial community composition are important to improve ruminant productivity.

Detoxification of ammonia through ureagenesis can occur as a response to greater concentrations of ammonia produced by excess RDP or amino acids supplied by MCP [6]. To improve and stabilize the growth rates of bacteria in the rumen, a concentration of 50 mg of ammonia N/L is considered the acceptable amount for ruminal bacteria [86]. However, ammonia detoxification may impact energy metabolism due to the increased energy cost of the process, resulting in negative implications on animal performance [6]. Issues such as degradation of the ruminal epithelium, hepatic toxicity, ketosis, pneumonia, mastitis, and laminitis are frequently associated with an imbalance in the energy-to-protein ratio [87].

### 4.4. Effects of Beta-Adrenergic Agonists on Protein, Supplemented Protein, and Nitrogen Utilization

Supplementation of *β*-AA in finishing beef cattle has been employed to increase growth, body weight before harvest, and carcass weight [88]. The addition of *β*-AA can increase lean muscle deposition by improving the utilization of amino acids for protein synthesis or by fluctuating the pattern of growth of the animal [89]. Additionally, serum urea concentration decreased with *β*-AA supplementation [90]. In beef cattle, supplementation of RAC with optimal concentrations of protein in the diet altered microbial ammonia, suggesting RAC can affect the breakdown of amino acids into ammonia by microorganisms in the rumen and impact ruminal degradation of dietary protein [91]. These results suggest an alternative option to increasing NPN in the diet as a source of ammonia, using additional true protein to establish optimal ruminal fermentation in finishing cattle diets [91]. Together, these data suggest *β*-AA alters processes in finishing cattle, such as N retention and urea recycling [91]. Further, the use of *β*-AA to improve the utilization of amino acids for protein synthesis may be related to their receptors. The interaction between beta-adrenergic receptors and a beta-adrenergic agonist has the potential to enhance muscle hypertrophy by promoting increased blood circulation to the skeletal muscle. These hemodynamic responses contribute to the improved supply of vital energy sources (mitochondrial ATP) and essential substrates (amino acids) necessary for protein synthesis [29,92]. Additionally, adipose tissue might facilitate the transfer of non-esterified fatty acids out of adipose stores, thereby increasing lipid degradation [25]. The heightened blood flow also has the capacity to elevate heart rate and enhance the circulation of diverse endocrine hormones, including insulin—an anabolic hormone—which significantly influences muscle protein metabolism [43,93]. Nonetheless, the impact of beta-adrenergic agonists on blood flow is transient, suggesting that the mechanisms driving muscle hypertrophy are unlikely to be solely attributed to hemodynamic alterations [94].

Animals fed rumen-degradable forms of nitrogen increased the response of RAC, illustrating that the type of protein (RDP and RUP) provided to the rumen microbiota may be important for optimizing the response of RAC in finishing cattle [91] and supports a relationship between effects of *β*-AA and nitrogen source. Indeed, research conducted by Walker and colleagues [16] in 2006 revealed that the utilization of *β*-AA in ruminants that were provided with dietary crude protein fractions (RDP and RUP) resulted in heightened nitrogen retention and improved absorption of amino acids. Arranged as a 2 × 3 factorial, treatments were implemented by including 0 or 200 mg of ractopamine-HCl and a urea (688 g/d metabolizable protein), solvent soybean meal (761 g/d metabolizable protein), or expeller soybean meal (808 g/d metabolizable protein) protein supplement. The researchers postulated that *β*-AA, in particular, led to an augmented uptake of amino acids by peripheral tissues after absorption, as evidenced by the reduction in total alpha-amino N levels within the bloodstream [16]. Thus, understanding the influence of CP degradability and metabolizable protein in response to *β*-AA is critical for protein metabolism and synthesis. By understanding these interactions, insight will be gained to improve diet formulation, taking full advantage of the action of *β*-AA and management in the finishing cattle industry [3].

### 4.5. Effect of Beta-Adrenergic Agonist on the Bacteria Population

Beta-adrenergic agonists, as synthetic catecholamines, produce similar reactions compared to natural catecholamines, binding with beta-adrenergic receptors to increase lipolysis, gluconeogenesis, and glycogenolysis in adipose tissue and the liver [88]. Norepinephrine and epinephrine, which are natural catecholamines, have shown varied effects on bacteria, such as stimulating bacterial growth [95], increasing the population of Gram-negative bacteria in in vitro experiments [96], as well as modifying gut motility and secretory response [97,98]. Indeed, adrenergic agonists have the capacity to decrease the frequency and intensity of contractions in the rumen, influencing the digestion process of dietary nutrients carried out by the microbial community within the rumen [97,99,100]. This outcome is primarily associated with alpha-adrenergic agonists (α-AA), such as phentolamine, which exhibit an affinity for alpha-adrenergic receptors (α-AR). Upon binding of these α-AA to α-AR, these receptors induce an inhibitory response, leading to the release of acetylcholine from postganglionic nervous terminals in the parasympathetic system. Consequently, the sympathetic system’s reaction to these processes indirectly hinders gastrointestinal functions and motility [101]. Overall, observed effects of adrenergic agonists also include altered digestion of nutrients by ruminal microbes and impacts on eructation of gases from the rumen [99,100]. In an experiment with sheep, the amount of rumen glucose increased in response to *β*-AA when the animal consumed greater amounts of rapidly fermented carbohydrates, predisposing the animal to acidosis [102]. Interestingly, researchers have also aimed to determine the effect of synthetic catecholamines, such as RAC, on the gut microbiome of livestock. Those results demonstrated that in sheep with oral inoculation of *E. coli* O157:H7, RAC increased pathogen proliferation in the cecum, noting implications for animal health and the environment [103]. In addition, natural catecholamines increase the affinity of bacterial iron utilization, an essential mineral for bacterial growth [104]. Catecholamines increase Gram-negative bacteria in studies in vitro [96]. As some of the bacteria present in the rumen for fermentation processes are Gram-negative [91], it is possible that the reaction of RAC as a synthetic catecholamine could improve iron affinity to ruminal microbes and increase bacterial population growth, impacting the balance and diversity of the ruminal ecosystem.

## 5. Conclusions

The beef industry in the US faces the challenge of increasing the supply of high-quality protein while reducing negative environmental impacts and maintaining the enterprise economy. Further, increases in the feed efficiency of animals are necessary to achieve improved beef production with limited resources. The gastrointestinal tract of ruminants is capable of absorbing nutrients and producing energy dependent on microbial communities. Changes in diet are one of the most common factors that can impact the rumen microbial communities. Technologies, such as beta-adrenergic agonists (ractopamine hydrochloride), supplemented during the last weeks before harvest, have been developed to increase lean muscle deposition, improve gain, and enhance feed efficiency. Additionally, as beta-adrenergic compounds have been shown to improve nitrogen retention, they may also influence protein metabolism and shape rumen microbial communities. By understanding the response of the interaction among protein in different concentrations, *β*-AA, and the rumen microbiome, researchers can further improve efficiency and animal growth, providing the tools to create diets that can maximize the response of those components.

## Data Availability

No new data were created or analyzed in this study. Data sharing is not applicable to this article.
